# Supplementary material for: Sequence-Specific Features of Short Double-Strand, Blunt-End RNAs Have RIG-I- and Type 1 Interferon-Dependent or -Independent Anti-Viral Effects
Source: Viruses. 2022 Jun 28;14(7):1407. doi: 10.3390/v14071407 (PMC9322957; doi:10.3390/v14071407)
Supplement: Supplementary file 1 [file viruses-14-01407-s001.zip › viruses-1740661-supplementary.pdf]

Supplementary Materials

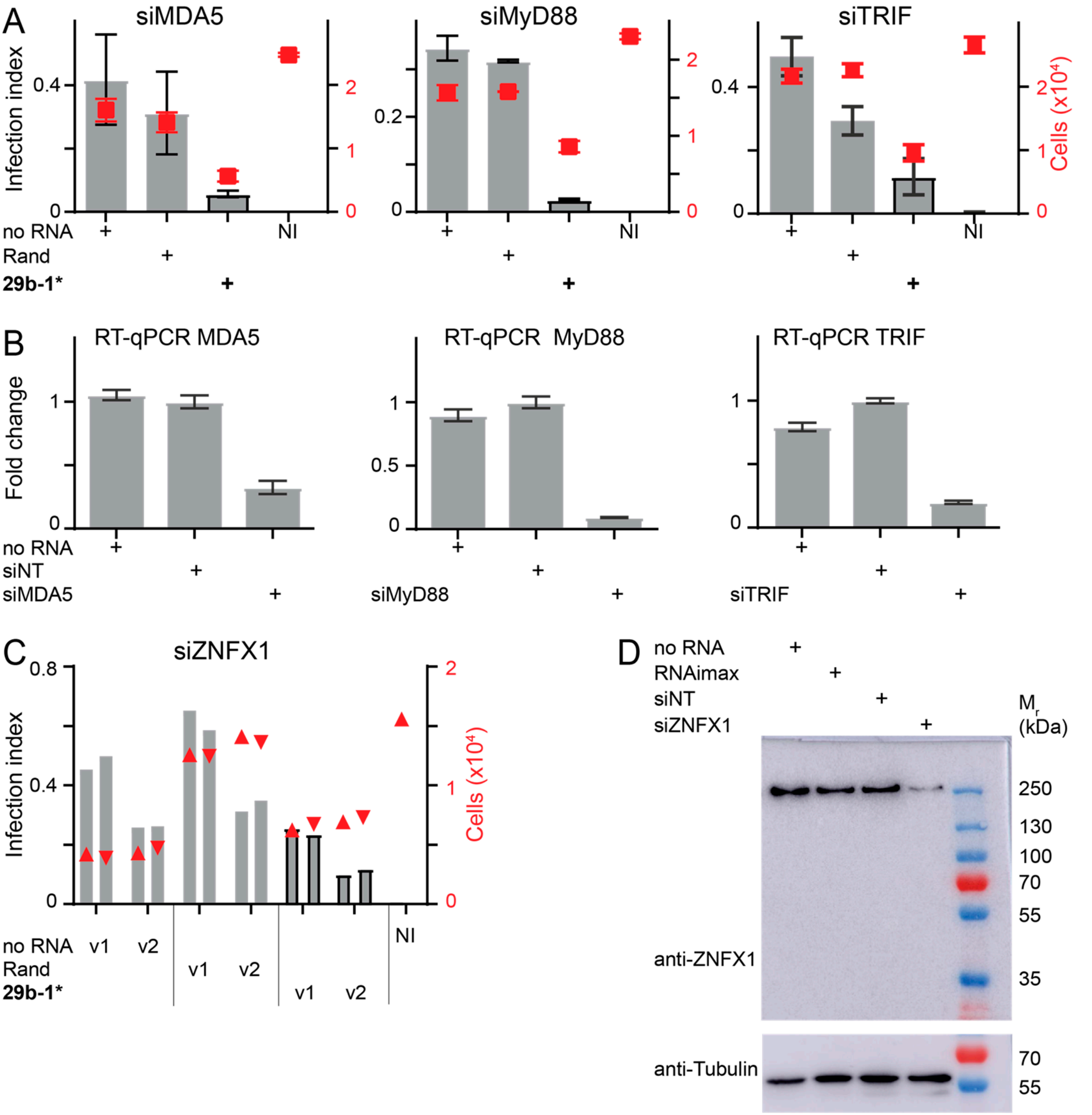

Supplementary Figure S1: No effects of MDA5, MyD88, TRIF, or ZNFX1 on anti-AdV-C5 effects by miR-29b-1* mimics.

A. siRNA-mediated knockdown of MDA5, MyD88, or TRIF in A549 cells has essentially no impact on the anti-viral effects of blunt-end miR29b-1* mimic (29b-1*) against AdV-C5. Infection index (left y-axis, bar plot) refers to the fraction of VI-positive cells over total number of cells analyzed (right y-axis, scatter plot). Shown are mean values from three technical replicates with standard deviation. No RNA refers to non-transfected control cells and Rand to transfection with a mimic with randomized miR29b-1* sequence. Rand and miR29b-1* mimics were obtained from Qiagen.

B. RT-qPCR control of mRNA knockdown levels by the siRNAs used.

C. No effect of siZNFX1 knockdown on AdV-C5 infection. Infection efficiency was scored as in (A). Two different two-fold dilutions of input virus were used and the two technical replicates are shown separately.

D. Control for ZNFX1 knockdown by the siRNAs used. Intracellular levels of ZNFX1 were analyzed by Western blot using a recombinant monoclonal rabbit anti-ZNFX1 antibody and a secondary HRP-conjugated anti-rabbit antibody. For a loading control, the blot was subsequently (without stripping) stained with a mouse monoclonal anti-alpha/beta tubulin antibody and a secondary HRP-conjugated anti-mouse antibody. Only the lower part of the membrane is shown for the loading control. The size of the molecular weight marker (M) protein bands are indicated. RNAimax refers to transfection with transfection reagent alone (no siRNA) and siNT refers to transfection with a control non-targeting siRNA.

Supplementary Table S1: List of dsRNA sequences (for simplicity, only one strand is shown).

Name	Supplier	sequence 5'-3'	comments	miR29b-1* base composition maintained
**miR29b-1***	Qiagen miScript	GCUGGUUUCAUAUGGUGGUUUAGA	unmodified, blunt-end dsRNA with 5'-OH and 3'-OH	yes
**miR29b-1***	Microsynth, custom-made	GCUGGUUUCAUAUGGUGGUUUAGA	unmodified, blunt-end dsRNA with 5'-OH and 3'-OH	yes
**miR29b-1* 3' overhangs**	Microsynth, custom-made	GCUGGUUUCAUAUGGUGGUUUAGA-UU	unmodified, dsRNA with 3' -UU overhangs, 5'-OH	no
**miR29b-1***	Dharmacon, miRIDIAN	GCUGGUUUCAUAUGGUGGUUUAGA	3' overhangs, monophosphate in the 5' antisense strand, proprietary ON-TARGET modifications on the passenger strand	yes
**RandmiR29b-1***	Qiagen custom-made miScript	UUUAUGCGGGGUGUCGUAUAAUGU	unmodified, blunt-end dsRNA with 5'-OH and 3'-OH	yes
**RandmiR29b-1***	Microsynth, custom-made	UUUAUGCGGGGUGUCGUAUAAUGU	unmodified, blunt-end dsRNA with 5'-OH and 3'-OH	yes
**miR29b-1*_sAU2**	Microsynth, custom-made	GCUGGUUUCAGUAGUUGGUUUAGA	unmodified, blunt-end dsRNA with 5'-OH and 3'-OH	yes
**miR29b-1*_sAU3**	Microsynth, custom-made	GCUGGUUUCAGUGAGUUGUUUAGA	unmodified, blunt-end dsRNA with 5'-OH and 3'-OH	yes
**scr1**	Qiagen custom-made miScript	UUUAUGCGCUUGAAUGUUGGGUAG	unmodified, blunt-end dsRNA with 5'-OH and 3'-OH	yes
**scr2**	Qiagen custom-made miScript	CUUAUGCGUUAAGUUGGUUGGUGA	unmodified, blunt-end dsRNA with 5'-OH and 3'-OH	yes
**scr3**	Qiagen custom-made miScript	UUUAUGCGUGUGGUCUGAUAAUGG	unmodified, blunt-end dsRNA with 5'-OH and 3'-OH	yes
**scr4**	Qiagen custom-made miScript	GUUAUGCGUUGUAGUUGUGGAAUC	unmodified, blunt-end dsRNA with 5'-OH and 3'-OH	yes
**scr5**	Qiagen custom-made miScript	CUUAUGCGAUUGGUUUGUGGAGUA	unmodified, blunt-end dsRNA with 5'-OH and 3'-OH	yes
**scr6**	Qiagen custom-made miScript	UUUAUGCGUCGGGUGUGUUGUAAA	unmodified, blunt-end dsRNA with 5'-OH and 3'-OH	yes
**scr7**	Qiagen custom-made miScript	CUUAUGCGAAUGGUGUGGUUAGUU	unmodified, blunt-end dsRNA with 5'-OH and 3'-OH	yes
**scr8**	Qiagen custom-made miScript	GUUAUGCGUCGUUGAGUAGUGUUA	unmodified, blunt-end dsRNA with 5'-OH and 3'-OH	yes
**scr9**	Qiagen custom-made miScript	GUUAUGCGUGUUGUCGGAUGUAUA	unmodified, blunt-end dsRNA with 5'-OH and 3'-OH	yes
**scr10**	Qiagen custom-made miScript	GUUAUGCGUUUCUGAUGAUGGAUG	unmodified, blunt-end dsRNA with 5'-OH and 3'-OH	yes
**scr11**	Qiagen custom-made miScript	GUUAUGCGUGUGGGACGAUUUUUA	unmodified, blunt-end dsRNA with 5'-OH and 3'-OH	yes
**scr12**	Qiagen custom-made miScript	UUUAUGCGGUAGUGAUCUGUAUGG	unmodified, blunt-end dsRNA with 5'-OH and 3'-OH	yes
**scr13**	Qiagen custom-made miScript	UUUAUGCGGAUGUGUAGGACUGUU	unmodified, blunt-end dsRNA with 5'-OH and 3'-OH	yes

**scr14**	Qiagen custom-made miScript	AUUAUGCGGAUGUGUGAGGUUUCU	unmodified, blunt-end dsRNA with 5'-OH and 3'-OH	yes
**scr15**	Qiagen custom-made miScript	GUUAUGCGUCCCUGGGUGUCGGUC	unmodified, blunt-end dsRNA with 5'-OH and 3'-OH	no
**scr16**	Qiagen custom-made miScript	UUUAUGCGUCCAUCCCCUCUGGCC	unmodified, blunt-end dsRNA with 5'-OH and 3'-OH	no
**scr17**	Qiagen custom-made miScript	AUUAUGCGCCUGGGGGCGGACGGU	unmodified, blunt-end dsRNA with 5'-OH and 3'-OH	no
**scr18**	Qiagen custom-made miScript	GUUAUGCGUUUGUUGCUAUGAUUU	unmodified, blunt-end dsRNA with 5'-OH and 3'-OH	no
**scr19**	Qiagen custom-made miScript	GUUAUGCGACGUGGGUCACAUCCG	unmodified, blunt-end dsRNA with 5'-OH and 3'-OH	no
**scr20**	Qiagen custom-made miScript	UUUAUGCGUUUUGGGGGUAAGACU	unmodified, blunt-end dsRNA with 5'-OH and 3'-OH	yes
**scr21**	Qiagen custom-made miScript	GUUAUGCGGAAGUUUGUGUCAUUG	unmodified, blunt-end dsRNA with 5'-OH and 3'-OH	yes
**scr22**	Qiagen custom-made miScript	UUUAUGCGGUAGUGAUCUGUAUGG	unmodified, blunt-end dsRNA with 5'-OH and 3'-OH	yes
**scr23**	Qiagen custom-made miScript	UUUAUGCGGAUGUGUAGGACUGUU	unmodified, blunt-end dsRNA with 5'-OH and 3'-OH	yes
**scr24**	Qiagen custom-made miScript	AUUAUGCGGAUGUGUGAGGUUUCU	unmodified, blunt-end dsRNA with 5'-OH and 3'-OH	yes
**scr25**	Qiagen custom-made miScript	AUUAUGCGUGGUGCUAGGAUUGUU	unmodified, blunt-end dsRNA with 5'-OH and 3'-OH	yes
**scr26**	Qiagen custom-made miScript	AUUAUGCGUGGUGUAAGUUGGCUU	unmodified, blunt-end dsRNA with 5'-OH and 3'-OH	yes
**scr27**	Qiagen custom-made miScript	UUUAUGCGUGUCGUGGAUGUGAAU	unmodified, blunt-end dsRNA with 5'-OH and 3'-OH	yes
**scr28**	Qiagen custom-made miScript	GUUAUGCGUCCCUGGGUGUCGGUC	unmodified, blunt-end dsRNA with 5'-OH and 3'-OH	no
**scr29**	Qiagen custom-made miScript	GUUAUGCGCCGACCCGGCCCUUCG	unmodified, blunt-end dsRNA with 5'-OH and 3'-OH	no
**scr30**	Qiagen custom-made miScript	AUUAUGCGCCUGGGGGCGGACGGU	unmodified, blunt-end dsRNA with 5'-OH and 3'-OH	no
**scr31**	Qiagen custom-made miScript	GUUAUGCGUUUGUUGCUAUGAUUU	unmodified, blunt-end dsRNA with 5'-OH and 3'-OH	no
**scr32**	Qiagen custom-made miScript	GUUAUGCGACGUGGGUCACAUCCG	unmodified, blunt-end dsRNA with 5'-OH and 3'-OH	no
**scr33**	Qiagen custom-made miScript	UUUAUGCGAUCGUGACAGCGCCCU	unmodified, blunt-end dsRNA with 5'-OH and 3'-OH	no

Supplementary Table S2: List of top 50 significantly upregulated and downregulated host genes upon dsRNA miR29b-1* mimic transfection (72h) (FDR < 0.05).

Upregulated host genes

GeneID	Log_2Fold change	FDR_pValue	Gene name	Gene_description
6352	11.794	9.82E-07	CCL5	chemokine (C-C motif) ligand 5
282617	10.63	7.20E-08	IL28B	interleukin 28B (interferon, lambda 3)
3429	10.239	3.21E-07	IFI27	interferon, alpha-inducible protein 27
54739	10.002	1.35E-06	XAF1	XIAP associated factor 1
6288	9.905	9.01E-07	SAA1	serum amyloid A1
4939	9.556	5.51E-07	OAS2	2'-5'-oligoadenylate synthetase 2, 69/71kDa
6373	9.338	1.57E-07	CXCL11	chemokine (C-X-C motif) ligand 11
684	9.2	1.57E-07	BST2	bone marrow stromal cell antigen 2
8638	9.197	1.35E-06	OASL	2'-5'-oligoadenylate synthetase-like
51296	9.055	1.33E-05	SLC15A3	solute carrier family 15, member 3
4599	8.948	9.85E-07	MX1	myxovirus (influenza virus) resistance 1, interferon-inducible protein p78 (mouse)
10964	8.906	2.82E-06	IFI44L	interferon-induced protein 44-like
3620	8.901	1.00E-05	IDO1	indoleamine 2,3-dioxygenase 1
8519	8.87	9.85E-07	IFITM1	interferon induced transmembrane protein 1 (9-27)
10537	8.61	3.65E-05	UBD	ubiquitin D
3456	8.564	1.57E-07	IFNB1	interferon, beta 1, fibroblast
27074	8.552	3.15E-06	LAMP3	lysosomal-associated membrane protein 3
4600	8.507	1.11E-06	MX2	myxovirus (influenza virus) resistance 2 (mouse)
64108	8.407	2.50E-06	RTP4	receptor (chemosensory) transporter protein 4
282616	8.39	3.54E-06	IL28A	interleukin 28A (interferon, lambda 2)
3437	8.375	7.06E-07	IFIT3	interferon-induced protein with tetratricopeptide repeats 3
3627	8.347	1.57E-07	CXCL10	chemokine (C-X-C motif) ligand 10
3433	8.342	8.23E-07	IFIT2	interferon-induced protein with tetratricopeptide repeats 2
282618	8.293	2.77E-06	IL29	interleukin 29 (interferon, lambda 1)
91543	8.246	1.57E-07	RSAD2	radical S-adenosyl methionine domain containing 2
51513	8.194	5.81E-06	ETV7	ets variant 7
9636	7.796	1.71E-07	ISG15	ISG15 ubiquitin-like modifier
6866	7.715	1.03E-06	TAC3	tachykinin 3
10346	7.372	9.85E-07	TRIM22	tripartite motif containing 22
259307	7.332	9.85E-07	IL4I1	interleukin 4 induced 1
629	7.312	1.85E-07	CFB	complement factor B
5655	7.235	7.79E-07	KLK10	kallikrein-related peptidase 10
2633	7.204	4.14E-06	GBP1	guanylate binding protein 1, interferon-inducible
115362	7.109	2.61E-05	GBP5	guanylate binding protein 5
2537	7.106	3.93E-06	IFI6	interferon, alpha-inducible protein 6
3434	7.008	9.01E-07	IFIT1	interferon-induced protein with tetratricopeptide repeats 1
8843	6.963	7.46E-06	HCAR3	hydroxycarboxylic acid receptor 3
27033	6.962	1.41E-05	ZBTB32	zinc finger and BTB domain containing 32
834	6.919	1.35E-06	CASP1	caspase 1, apoptosis-related cysteine peptidase (interleukin 1, beta, convertase)
3665	6.861	4.88E-07	IRF7	interferon regulatory factor 7
84941	6.801	2.86E-07	HSH2D	hematopoietic SH2 domain containing
1440	6.781	1.35E-06	CSF3	colony stimulating factor 3 (granulocyte)
6289	6.718	9.85E-07	SAA2	serum amyloid A2
10561	6.699	8.53E-06	IFI44	interferon-induced protein 44
116071	6.576	7.83E-07	BATF2	basic leucine zipper transcription factor, ATF-like 2
10866	6.372	1.00E-06	HCP5	HLA complex P5 (non-protein coding)
6364	6.258	0.000387248	CCL20	chemokine (C-C motif) ligand 20
7318	6.235	6.29E-06	UBA7	ubiquitin-like modifier activating enzyme 7
390035	6.191	4.00E-07	OR52K3P	olfactory receptor, family 52, subfamily K, member 3 pseudogene
6348	6.158	9.60E-05	CCL3	chemokine (C-C motif) ligand 3

Downregulated host genes

GeneID	Log_2Fold change	FDR_pValue	Gene name	Gene_description
25840	-5.731	5.34E-05	METTL7A	methyltransferase like 7A
4887	-5.495	1.35E-06	NPY2R	neuropeptide Y receptor Y2
6424	-5.304	0.000221921	SFRP4	secreted frizzled-related protein 4
7276	-5.221	1.48E-05	TTR	transthyretin
643763	-5.138	5.19E-06	UG0898H09	uncharacterized LOC643763
5579	-5.125	0.031115944	PRKCB	protein kinase C, beta
23352	-4.83	7.67E-06	UBR4	ubiquitin protein ligase E3 component n-recognin 4
89919	-4.761	0.030886003	C14orf56	chromosome 14 open reading frame 56
124	-4.752	9.82E-07	ADH1A	alcohol dehydrogenase 1A (class I), alpha polypeptide
4916	-4.712	2.05E-05	NTRK3	neurotrophic tyrosine kinase, receptor, type 3
11013	-4.71	2.82E-06	TMSB15A	thymosin beta 15a
81578	-4.648	0.000242797	COL21A1	collagen, type XXI, alpha 1
126	-4.489	1.21E-05	ADH1C	alcohol dehydrogenase 1C (class I), gamma polypeptide
79170	-4.436	0.000296926	PRR15L	proline rich 15-like
90952	-4.432	0.000258277	ESAM	endothelial cell adhesion molecule
4129	-4.411	0.000100383	MAOB	monoamine oxidase B
7429	-4.323	3.36E-06	VIL1	villin 1
11240	-4.309	0.000142596	PADI2	peptidyl arginine deiminase, type II
155006	-4.264	0.029351193	TMEM213	transmembrane protein 213
5137	-4.208	3.92E-06	PDE1C	phosphodiesterase 1C, calmodulin-dependent 70kDa
54474	-4.106	0.000224118	KRT20	keratin 20
866	-4.09	1.15E-05	SERPINA6	serpin peptidase inhibitor, clade A (alpha-1 antiproteinase, antitrypsin), member 6
80119	-3.985	1.84E-06	PIF1	PIF1 5'-to-3' DNA helicase homolog (S. cerevisiae)
79733	-3.976	5.69E-05	E2F8	E2F transcription factor 8
3851	-3.932	1.84E-05	KRT4	keratin 4
221357	-3.887	4.44E-05	GSTA5	glutathione S-transferase alpha 5
147381	-3.886	6.98E-05	CBLN2	cerebellin 2 precursor
56172	-3.869	1.33E-05	ANKH	ankylosis, progressive homolog (mouse)
53335	-3.853	0.041900463	BCL11A	B-cell CLL/lymphoma 11A (zinc finger protein)
114800	-3.818	0.001756483	CCDC85A	coiled-coil domain containing 85A
100169890	-3.787	0.045634698	PEG3-AS1	PEG3 antisense RNA 1 (non-protein coding)
100506627	-3.784	0.000652152	DCDC5	doublecortin domain containing 5
246213	-3.722	0.033581658	SLC17A8	solute carrier family 17 (sodium-dependent inorganic phosphate cotransporter), member 8
10874	-3.721	5.92E-05	NMU	neuromedin U
93663	-3.679	2.33E-05	ARHGAP18	Rho GTPase activating protein 18
55789	-3.671	5.76E-05	DEPDC1B	DEP domain containing 1B
3026	-3.665	5.88E-06	HABP2	hyaluronan binding protein 2
167681	-3.639	1.61E-06	PRSS35	protease, serine, 35
90293	-3.632	0.000118498	KLHL13	kelch-like 13 (Drosophila)
57405	-3.61	2.67E-05	SPC25	SPC25, NDC80 kinetochore complex component, homolog (S. cerevisiae)
9723	-3.609	1.48E-05	SEMA3E	sema domain, immunoglobulin domain (Ig), short basic domain, secreted, (semaphorin) 3E
1272	-3.605	2.70E-05	CNTN1	contactin 1
440823	-3.592	0.000371116	MIAT	myocardial infarction associated transcript (non-protein coding)
143686	-3.566	1.12E-05	SESN3	sestrin 3
345557	-3.563	3.25E-05	PLCXD3	phosphatidylinositol-specific phospholipase C, X domain containing 3
8671	-3.548	1.58E-05	SLC4A4	solute carrier family 4, sodium bicarbonate cotransporter, member 4
91057	-3.528	0.000107083	CCDC34	coiled-coil domain containing 34
1428	-3.509	1.98E-05	CRYM	crystallin, mu
3109	-3.508	1.24E-05	HLA-DMB	major histocompatibility complex, class II, DM beta
7474	-3.501	6.52E-05	WNT5A	wingless-type MMTV integration site family, member 5A

Supplementary Table S3: List of significant-scoring pathway maps (p.value < 0-05) of upregulated and downregulated genes upon dsRNA miR29b-1* mimic (72h) transfection in A549 cells. Obtained from MetaCore enrichment analysis.

Upregulated

Maps	Total no of genes in the pathway	pValue	No of differentially upregulated genes from this pathway	list of differentially upregulated genes from the pathway
Immune response (*IFN alpha/beta signaling pathway*)	24	9.69E-06	10	PML, ISG15, IFI6, IFN-alpha, SOCS1, IRF2, IRF9, IRF1, IFN-beta, USP18
Immune response (*Role of PKR in stress-induced antiviral cell response*)	57	1.71E-04	14	p38alpha (MAPK14), TRAF3, ATF-2, TRAF2, IFN-alpha, IL-8, TRAF6, IRF7, IRF3, TRIF (TICAM1), IRF1, TLR3, IFN-beta, TNF-alpha
Innate immune response to viral infection	28	2.88E-04	9	TRAF3, IFN-alpha, IL-8, LGP2, IRF7, IRF3, TRADD, TLR3, IFN-beta
Immune response (*Classical complement pathway*)	53	1.08E-03	12	C5aR, C1r, C3, C3b, C3c, C2, iC3b, C3dg, C2b, C3a, C1s, C2a
Immune response (*IFN gamma signaling pathway*)	56	5.56E-03	11	CBP, VCAM1, ATF-2, SMAD7, SOCS1, IRF9, CSF1, IRF1, p300, p38 MAPK, AFAP
Immune response (*Antiviral actions of Interferons*)	52	9.35E-03	10	CBP, INDO, WARS, IFN-alpha, ADAR1, IRF9, IRF3, IRF1, p300, IFN-beta
Signal transduction (*NF-kB activation pathways*)	51	2.28E-02	9	Ubiquitin, c-IAP2, TRAF3, TRAF2, c-IAP1, TRAF6, TRADD, ZFP91, TNF-alpha

Downregulated

Maps	Total no of genes in the pathway	pValue	No of differentially upregulated genes from this pathway	list of differentially upregulated genes from the pathway
Cell cycle (*Chromosome condensation in prometaphase*)	21	3.97E-10	16	Cyclin B, Histone H3, CAP-G, E140CAP-G/G2, INCENP, CAP-C, CAP-D2/D3, Aurora-A, CAP-E, Condensin, CAP-H/H2, CNAP1, Aurora-B, TOP2, BRRN1, Histone H1
Cell cycle (*Start of DNA replication in early S phase*)	32	5.12E-08	18	MCM3, CDC7, ORC1L, MCM5, RPA3, MCM4, MCM10, ASK (Dbf4), CDC45L Geminin, CDC18L (CDC6), Cyclin E, RPA1, ORC5L, MCM4/6/7 complex, MCM2, HP1 alpha, Histone H1
Cell cycle (*Initiation of mitosis*)	26	4.36E-07	15	Cyclin B2, Histone H3, CDC25C, FOXM1, Cyclin H, Nucleolin, Wee1, Kinase MYT1, Lamin B, KNSL1,MAT1, AKT(PKB),Cyclin B1, PLK1,Histone H1
Cell adhesion (*Histamine H1 receptor signaling in the interruption of cell barrier integrity*)	45	6.56E-06	19	MLCP (cat), ZO-1, Beta-catenin, CPI-17, Alpha-catenin, Paxillin, IP3 receptor, ROCK, Calmodulin, PLC-beta, Vinculin, PKC-alpha, MLCK, Occludin, G-protein beta/gamma, Actin cytoskeletal, MELC, Cofilin p120-catenin
Cell adhesion (*Gap junctions*)	30	2.35E-03	11	Tubulin beta, Cingulin, Connexin 32, ZO-1, Connexin 50, Actin, Caveolin-1 Tubulin alpha, PKC, Occludin, Tubulin (in microtubules)
Cell cycle (*Regulation of G1/S transition*)	38	1.71E-02	11	TGF-beta 2, PP2A regulatory, CDC25A, SMAD2, CDK6, GSK3 beta, p70 S6 kinase1, Cyclin E, p16INK4, Skp2, TGF-beta receptor type I
Cell adhesion (*Integrin-mediated cell adhesion and migration*)	48	1.76E-02	13	MLCP (cat), DOCK1, MyHC, Paxillin, ROCK, alpha-10/beta-1 integrin, Vinculin, Actin cytoskeletal, Cofilin Collagen IV, MELC, MYLK1, MLCK

Supplementary Table S4: List of RT-qPCR primers.

	forward 5' - 3'	reverse 5' - 3'
**RIG-I**	TGCGAATCAGATCCCAGTGTA	TGCCTGTAACTCTATACCCATGT
**MAVS**	GGACGAAGTGGCCTCTGTCTA	CATGGGGTAACTTGGCTCCTT
**MDA5**	GGCACCATGGGAAGTGATT	ATTTGGTAAGGCCTGAG
**MyDD88**	CGCCTCTGTAGGCCGACTGC	CAGCCTCCTCCTGCTGCTGC
**TRIF**	GCCACCTTCTGCGAGGATTTC	GTTCACCTGGTGCAGGCTC
**EEF1A1**	agcaaaaatgacccaccaatg	ggcctggatggttcaggata
**TBP**	gcccgaaacgccgaatata	cgtggctctcttatcctcatga
**TFRC**	catttgtgagggatctgaacca	cgagcagaatacagccactgtaa
**ACTB**	ccaaccgcgagaagatga	ccagaggcgtacagggatag
**GAPDH**	agccacatcgctcagacac	gcccaatacgaccaaatcc
